# THE PRICE OF DEMENTIA: POLICY STRATEGIES TO NARROW HEALTH DISPARITY GAPS, REDUCE COSTS, AND IMPROVE QUALITY OF LIFE

**DOI:** 10.1093/geroni/igz038.1690

**Published:** 2019-11-08

**Authors:** Nora Super, Rajiv Ahuja, Kevin Proff

**Affiliations:** 1 Milken Institute Center for the Future of Aging, Washington, District of Columbia, United States; 2 Milken Institute, Washington, District of Columbia, United States; 3 Milken Institute, Los Angeles, California, United States

## Abstract

Historically, a diagnosis of Alzheimer’s disease and related dementias (ADRDs) created a sense of hopelessness among those diagnosed, their families, and physicians. The misperception that there is no available medical treatment or interventions inhibited patients from receiving cognitive assessments or exploring home- and community-based interventions that could improve quality of life. Today, researchers have developed evidence-based pathways to improve brain health, reduce dementia risk, and enhance lifelong cognitive function. This report highlights recent scientific advancements focused on medical and lifestyle interventions that reduce the risk of ADRDs and slow the disease progression. To improve the current system and reduce the risk of dementia and improve brain health, the following policy areas are recommended: 1) promote brain health strategies to delay onset and reduce the stigma associated with cognitive decline; 2) increase early detection and cognitive screening efforts; 3) build a dementia-capable workforce; 4) create seamless transitions from health systems to community-based services to better support those with dementia; 5) improve caregiver training and support; and 6) increase funding for research related to ADRDs. Understanding that ADRDs disproportionately impact certain populations, these policy recommendations include strategies to narrow health disparities. Previous Milken Institute research highlighted the disproportionate health impacts of dementia on women, which costs the economy trillions of dollars. This report expands on that analyses and stratifies the data by race and income as well. Ensuring that these policy recommendations address disproportionately impacted groups will benefit the broader healthcare system and alleviate the burden felt by all patients and families.

